# What the CERAD Battery Can Tell Us about Executive Function as a Higher-Order Cognitive Faculty

**DOI:** 10.1155/2010/510614

**Published:** 2010-05-26

**Authors:** Rochelle E. Tractenberg, Gerda Fillenbaum, Paul S. Aisen, David E. Liebke, Futoshi Yumoto, Maragatha N. Kuchibhatla

**Affiliations:** ^1^Departments of Neurology Biostatistics, Bioinformatics & Biomathematics, and Psychiatry, Georgetown University School of Medicine, Washington, DC 20057, USA; ^2^Collaborative for Research on Outcomes and Metrics, Georgetown University Medical Center, Washington, DC 20057, USA; ^3^Department of Psychiatry and Behavioral Sciences, Center for the Study of Aging and Human Development, Duke University Medical Center and Geriatric Research, Education and Clinical Center, Veterans Administration Medical Center, Durham, NC 27710, USA; ^4^Department of Neurology, University of California, San Diego, La Jolla, CA 92093, USA; ^5^Department of Measurement, Statistics and Evaluation, University of Maryland, College Park, MD 20742, USA; ^6^Department of Biostatistics and Bioinformatics, Duke University Medical Center, Durham, NC 27710, USA

## Abstract

Executive function (EF) is believed to control or influence the integration and application of cognitive functions such as attention and memory and is an important area of research in cognitive aging. Recent studies and reviews have concluded that there is no single test for EF. Results from first-order latent variable modeling have suggested that little, if any, variability in cognitive performance can be directly (and uniquely) attributed to EF; so instead, we modeled EF, as it is conceptualized, as a higher-order function, using elements of the CERAD neuropsychological battery. Responses to subtests from two large, independent cohorts of nondemented elderly persons were modeled with three theoretically plausible structural models using confirmatory factor analysis. Robust fit statistics, generated for the two cohorts separately, were consistent and support the conceptualization of EF as a higher-order cognitive faculty. Although not specifically designed to assess EF, subtests of the CERAD battery provide theoretically and empirically robust evidence about the nature of EF in elderly adults.

## 1. Introduction

Executive function (EF) has become an area of great interest to researchers in cognitive psychology and cognitive aging especially [1–3]. In 2003 the National Institutes of Health held a 2.5-day trans-NIH workshop focused on the construct and its study, and the Committee on Research of the American Neuropsychiatric Association recently summarized a variety of issues in the study and understanding of EF that should be pursued and prioritized in future research [4]. Cognitive aging is a critical area of research [5] and EF is important in cognitive aging either as a cause of decline associated with aging or as an indicator of this decline that is not unique (see [2], for review). 

In his recent survey of the literature, Salthouse [6] explored the range of definitions of EF in a series of articles from 1994 through 2004. Definitions and assessments of EF vary (see [6–8]), but it is generally accepted that it involves control of the integration and application of cognitive functions. That is, in spite of disagreement and uncertainty about a specific definition of EF, there is widespread agreement that it is a “higher-order” cognitive function. However, as reviewed by Royall et al. [4], studies of the “structure” of EF have tended to emphasize first-order structures (e.g., by exploratory factor analysis; see pages 388–390, Table 3). 

Many recent studies and reviews of the literature have concluded that there is no single test for EF (see [1–3] for reviews of EF dimensions and tests), and results from latent variable modeling have suggested that little, if any, variability in cognitive performance can be directly attributed to EF [2, 6, 9]. Although latent variable modeling (structural equation modeling, and/or confirmatory factor analysis) has been employed in the most recent studies of EF and its makeup, no model has tested hypotheses about EF as a higher-order factor—the statistical representation of a cognitive function that may be directly measured but is also measured through its influence on lower-order, or more fundamental, functions such as memory and attention.

The present study had two purposes. The first was to determine whether, given an array of measures selected to assess diverse aspects of cognitive function, evidence can be found to support the conceptualization of EF as a higher-order cognitive function in elderly persons without dementia. The second purpose was to replicate such evidence, if possible, in two independent cohorts with the same battery of cognitive tests.

The measures in the neuropsychological battery of the Consortium to Establish a Registry for Alzheimer's Disease (CERAD) [10] were selected for that project to assess those areas of cognition particularly affected in Alzheimer's disease. As such, no measures specifically designed to assess EF were included; however, some of the measures present (e.g., Verbal Fluency, reverse spelling of WORLD in the Mini-Mental State Exam [11]) are representative of tasks proposed as measures of EF [3, 12]. Since there is no one-to-one correspondence between a specific task and a specific neuropsychological function [12], but rather, some measures are more strongly oriented to a particular cognitive function than others, the tasks in the CERAD battery, designed to assess a broad array of cognitive functions, offer an appropriate set for the purposes of this study, which are to seek statistical evidence of EF as a higher-order function and evaluate the consistency of this evidence in independent cohorts.

The analytic approach was to build a model EF as it is conceptualized, namely, as a higher-order function. Instead of simply estimating the fit of this particular model to the data, two theoretically plausible alternative models were also fit [13], so that the fit of this higher-order model could be compared to that of a model where EF was not a higher-order factor but was instead one of a set of correlated factors, and with a model with a single factor to explain the covariance among all test scores. The modeling was replicated in two large and independent cohorts of elderly persons, and fit statistics were computed to provide evidence of whether a higher-order model of EF is a productive element to incorporate into our evolving conceptualization of this construct.

## 2. Methods

### 2.1. Subjects

Two independent community-based cohorts of elderly individuals were assessed with the CERAD battery, among other tests, during the period 1987–1999.

**Cohort 1**

CERAD control subjects (*N* = 460). CERAD consisted of a consortium of Alzheimer Disease Research Centers (ADRCs) funded by the National Institute on Aging. Each of the 24 participating ADRCs was invited to submit information based on CERAD materials for 40 patients with Alzheimer's disease, and 30 control subjects, 50 years of age and older, assessed as cognitively normal, ambulatory, without conditions that could affect cognition, and who were not kin to an ADRC patient with AD. Participation in CERAD was approved by the IRBs at each participating site and signed consents were obtained.

**Cohort 2**

EPESE participants (*N* = 401). Duke EPESE is one of five EPESE sites that carried out longitudinal studies funded by the National Institute on Aging to determine the health status, change in health status, and health service use of persons 65 years of age and older. Data were gathered from a stratified random household sample (*N* = 4,162; 80% response rate) in five counties (one primarily urban, four primarily rural) in the piedmont area of North Carolina. Blacks were deliberately oversampled and represent 54% of the participants [14]. A stratified subsample of the EPESE cohort participated in a study of the incidence and prevalence of dementia [15]. While the EPESE dementia study subsample included 458 participants with normal cognition, information on the CERAD battery could only be obtained from 401 of these because of relocation, inability to find individuals, death, and/or poor health which precluded ability to respond to the CERAD battery, and in some cases, unwillingness to do so. Both Duke EPESE and the dementia study were approved by the Duke IRB, and signed consents were obtained.The same criteria were used in both the CERAD and EPESE cohorts to determine the absence of dementia, and the same procedures were used to train, administer, and score the measures of the CERAD neuropsychological battery (detailed in [10]). Only data from the baseline evaluation of all subjects with the consensus “diagnosis” of cognitively normal were included in the present study.

### 2.2. Materials

The CERAD battery [10, 16] includes the measures described below, presented in the order indicated. Another measure, Word List Recognition (recognition of the original 10 words presented in the Word List Learning task, when embedded in 10 new words), was considered for our analyses, but not included, since people who are cognitively intact make few errors on this task (data not shown; see also [17]).

***Verbal Fluency* (VERBFU_T)**

The number of (unique) animals that can be named within 60 seconds. Scoring range is 0 on up.

***15-Item Boston Naming Test* (NBOSTOT) [18]**

15 of the 60 items of the Boston Naming Test were selected so that they represent words of high, medium, and low frequency in the English language. Scoring range is 0–15.

***Mini-Mental State Examination* (MMSE) (NMMSE_TOT) [11]**

A brief screen of cognitive function in which spelling WORLD backwards is used instead of the serial seven subtraction item. Scoring range is 0–30. The MMSE can be considered to tap many different cognitive domains [19]; in the present samples, particularly in CERAD where cognitively normal people made few errors, it is likely to have measured mainly differences in score on WORLD backwards, that is, what is usually called “concentration”. Because of the multidimensionality of the MMSE total score, the two 3-factor models were fit separately in each cohort with the MMSE score coded in one of three ways; (a) total MMSE score treated as an indicator of “EF”; (b) separated into two scores: score on WORLD backwards (treated as an indicator of “EF”), and score on the remaining items (not treated as an indicator of “EF”); and (c) only WORLD backwards score (treated as an indicator of “EF”).

***10-Item Word List Learning Task *(NWRDLSTME)**

Ten common nouns presented consecutively and read aloud by the participant (or read to, and repeated by, the participant if the participant cannot read), with a different order used on each of three successive occasions. After each of the three occasions the participant is asked to recall the nouns that he or she had read. Scoring range is 0–10 for each presentation, or, as used here, 0–30 for all three presentations combined.

***Constructional Praxis* (NCIRCLE, NDIAMOND, NRECTNGL, NCUBE) [20]**

Copying a circle, diamond, overlapping rectangles, and a cube, and each can be scored separately, and summed scores can range from 0 to 11.

***Word List Recall* (NWRDLST4)**

It is a delayed recall of the nouns of the 10-item Word List Learning task. Scoring range is 0–10.

### 2.3. Statistical Methods

 Confirmatory factor analyses (CFAS) were carried using EQS 6.1 (Multivariate Software, Inc., 2005). EQS computes robust fit statistics reflecting multiple dimensions of the model-data fit (i.e., not simply a chi-square statistic for model fit). Fit indices describing the appropriateness of the model given the data (described below) were recorded for each model run separately for each cohort. Models were fit using robust methods (i.e., methods that are appropriate when modeling assumptions are not met). In all models, the same observed (indicator) variables appear in the same order. 

#### 2.3.1. Model Fit

The models (one-factor (null), three correlated (first-order) factors, EF as higher-order factor) were fit separately to the data from each cohort's baseline visits. Five different aspects of fit were assessed for each run in each cohort, reflecting general data-model fit (Satorra-Bentler model chi square, *χ* ^2^—lower is better), assessment of the fit of the model to data in hypothetical replications (Akaike's Information Criterion, AIC—lower is better), incremental model fit relative to an independence model (comparative fit index, CFI—between 0.95 and 1.0 is desirable), error in approximation of the data by the model (root mean square error of approximation, RMSEA—smaller and upper bound of 90% CI <0.06 is ideal), and the mean absolute value of the covariance residuals (standardized root mean square residual, SRMR—smaller and <0.09 is best) (criteria for fit indices are based on standard, and not robust, versions; see [22]). These indices describe different aspects of the fit of the model; we would consider a model that is superior in all indices to be the “best fitting”. Robust versions of all fit statistics were computed except for the SRMR, which has no robust counterpart but which summarizes the fit in a way the other (robust) indices do not.

**Figure 1:**
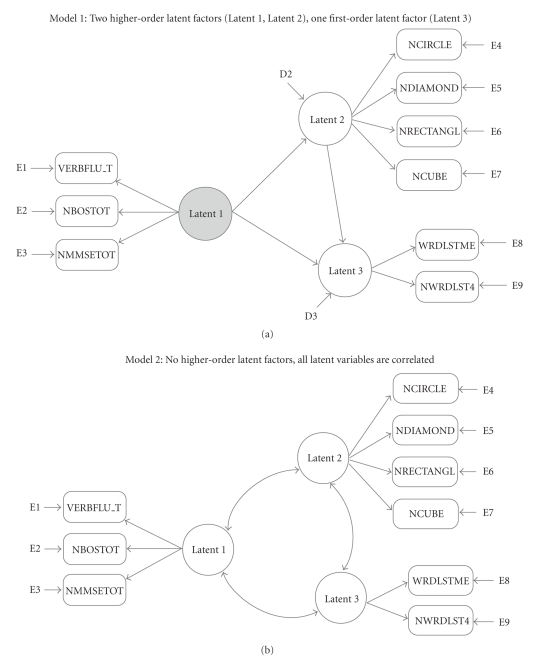
Confirmatory factor models with nine variables included. The total MMSE is shown in the models below, but we also fit the three models with WORLD backwards on the factor with verbal fluency and the Boston Naming Task—with and without the remainder of the MMSE on the “praxis” factor. The one-factor (null) model is not shown, but was also fit with the total MMSE; with the MMSE separated into score on WORLD and score on the remainder of the MMSE; and with just the WORLD backwards score.

Support for the same model was sought from all indices (consistency) as well as within both cohorts (replicability). We went through the modeling procedures three times, obtaining fit statistics for all when the MMSE was included as a total MMSE score (on the “EF” factor, when appropriate), a WORLD backwards score (on the “EF” factor, when appropriate) and the remainder of the MMSE total score (on the “praxis” factor, when appropriate), and a WORLD backwards score (on the “EF” factor, when appropriate) without the remainder of the MMSE total score. We fit two multifactor models: one hypothesizing causal (Model 1), and one correlational (Model 2) relations in the structural model. These are shown in Figure 1. Model 3, a “null” model (not shown in Figure 1), specified all scores loading on a single latent factor. In this manner we were able to examine the fit of each model relative to the fit statistics as well as relative to reasonable alternative models [13].

## 3. Results

### 3.1. Sample Characteristics

Table 1 presents the demographic and test performance summary statistics for the two cohorts. The groups were significantly different in terms of nearly all test scores and key demographic characteristics (age, education, racial makeup). 

The CERAD study cohort was younger, had more education, and scored significantly higher on all but one of the nine tests that were analyzed in these models (all *P* < .01 after Bonferroni adjustment for 15 tests). This cohort was 93% white, compared with 40% white in the EPESE cohort (*P* < .05), but the two groups had similar proportions of women (66% in CERAD and 62% in EPESE).

### 3.2. Structural Equation Modeling/CFA

Three measurement models were selected on the basis of theoretical considerations and additional exploratory analyses that are described briefly in the appendix, Boston Naming (BN), MMSE total, or WORLD backwards when this was separated from the MMSE total, and verbal fluency constituted one latent factor (Latent 1), which we generally characterized as representing “executive function” (“EF”)—although *all* the scores were selected for their potential as EF indicators. The other measurement models (“Latent 2” and “Latent 3”) were reflective of more specific domains (i.e., memory and praxis). The four constructional praxis scores (rectangle, cube, circle, diamond) constituted the “praxis” latent variable, and when WORLD backwards was separated from the remainder of the MMSE score, this MMSE-remainder was combined with the praxis scores. The two memory scores were combined to represent a “memory” latent variable; because the MMSE component items are broader than these two memory tests, we chose to combine the remainder MMSE score with the other latent variable (praxis) when those analyses were run.

Preliminary exploratory modeling (see appendix) supported the same measurement models (i.e., latent variable with associated observed scores) for both cohorts; so the CFA models that we fit were also the same in both cohorts. In addition to the two 3-factor structural models described above, we also obtained fit statistics reflecting a one-factor model of EF. That is, we selected the nine tests as potential indicators of EF; so a one-factor model of EF with all scores as indicators was also fit in each cohort. Thus, a total of three structural models were fit. Model 1 is consistent with a higher-order conceptualization of EF while Model 2 is inconsistent with this conceptualization and is consistent with the ways in which EF is typically modeled, although inconsistent with the theoretical representation of EF as a higher-order faculty. Model 3, a single-factor model for all of the scores we analyzed (not shown in Figure 1), is also inconsistent with a higher-order conceptualization.

The fit statistics for the three models, fit separately in each cohort and run three times with the different MMSE configurations, are compiled in Table 2 for the three structural models that we estimated using the total MMSE score, the WORLD backwards and remaining MMSE score on separate latent variables, and only the WORLD backwards score on the “EF” latent variable, as described above.

In Table 2 it can be seen that the robust statistic for model fit (Satorra-Bentler *χ* ^2^) reflects good fit of Model 1 (EF as higher-order factor) to the data in both cohorts (both *P* > .99). Model 2 (EF as first-order factor) reflects moderate fit to the EPESE data (*P* = .10) but not for the CERAD data (*P* = .009). Similarly, the other four fit statistics suggest that the model that includes a higher-order factor (Model 1) fits better than the first-order model (Model 2), and this is true for all indices, and both cohorts, across the three MMSE configurations. Model 3, the one-factor model, hypothesizing that all nine measures represent a single underlying latent factor, failed to meet any robust fit index criterion except SRMR (i.e., fit poorly in both cohorts). 

Model 1 (EF as higher-order factor) was the best supported in both cohorts, irrespective of how the MMSE was included. When we compared the fit statistics of Model 1 across the three configurations of MMSE score, we found that when the total MMSE score was included as an indicator of EF (first run, shown at the top of Table 2), the differences between the higher-order (Model 1) and first-order (Model 2) configurations were more striking, in terms of model fit (Satorra-Bentler *χ* ^2^), which was good for Model 1 (both *χ* ^2^ < 9, both *P* > .99), but poor (CERAD: *χ* ^2^ = 43.27, *P* < .001) or marginal (EPESE: *χ* ^2^ = 32.96, *P* = .10) for Model 2. Similar differences are observed, although difficult to interpret, in AIC. CFI, SRMR, and RMSEA did not differentiate between Models 1 and 2 when total MMSE was included on the EF factor, although CFI and RMSEA values were better for Model 1 than for Model 2. 

When WORLD backwards was modeled on the EF factor and the remainder of the MMSE total score was modeled on the “Praxis” factor (second run, middle of Table 2), the differences between the higher-order (Model 1) and first-order (Model 2) configurations were moderate. In terms of model fit (Satorra-Bentler *χ* ^2^), Model 1 (*χ* ^2^ = 18.91, *P* = .94) but not Model 2 (*χ* ^2^ = 65.13, *P* < .001) was a good fit in the CERAD cohort, but a poor fit in the EPESE cohort (both *χ* ^2^ > 50, both *P* ≤ .001). AIC supported Model 1 over Model 2 in both cohorts, and CFI supported Model 1 over model 2 in the CERAD, but not the EPESE cohort. As when total MMSE was modeled, SRMR, and RMSEA did not differentiate between Models 1 and 2 when WORLD-backward was included on the EF factor and the remainder of the MMSE score was modeled on the “Praxis” factor, although CFI, SRMR and RMSEA values were all better for Model 1 than for Model 2. 

When WORLD backwards alone was modeled (last run, bottom of Table 2), the differences between the higher-order (Model 1) and first-order (Model 2) configurations were much less striking, in terms of model fit (Satorra-Bentler *χ* ^2^) and AIC. As when total MMSE was modeled, CFI, SRMR, and RMSEA did not differentiate between Models 1 and 2 when WORLDbackward was included on the EF factor and the remainder of the MMSE score was excluded from the model, although CFI and RMSEA values were better for Model 1 than for Model 2. 

Irrespective of the representation of the MMSE across our models, Model 1, hypothesizing EF as a higher-order factor, was best supported (except by SRMR). The one-factor (null) model was not a good fit to the data in either cohort. Although the MMSE is a multidimensional test, the clearest distinctions between the models were obtained when the total MMSE score was hypothesized to be an indicator of EF (first run). We do not claim that the models we fit are “true”, but it is useful to examine the model-estimated relationships between the variables, in the two cohorts, under Model 1. The standardized pathweights for Model 1, with MMSE total score hypothesized as an EF, appear in Table 3. The estimated variability (*R* ^2^) in each observed score that is explained by its hypothesized associated latent variable is included, separately for each cohort. Standardized pathweights can be interpreted similar to regression coefficients, and more important for our purposes is that the pattern in the *R* ^2^ values is quite similar for the two cohorts.

In the first row of Table 3 it can be seen that the hypothesized underlying latent factor (“memory”) explains 80.4% of the variability in the sum of 3 trials memory score in the EPESE cohort. Similarly, in the CERAD cohort, 71.5% of the variability in sum of 3 trials performance is explained by the same latent variable. The pathweights and associated *R* ^2^ values are very similar for the two cohorts with two exceptions, both in the “Praxis” latent variable. The pathweights for circle and rectangle are not significant for the CERAD cohort, but they are for the EPESE cohort. This may be due to lower levels of variability on these scores in the CERAD relative to the EPESE cohort (see Table 1).

## 4. Discussion and Conclusions

We analyzed nine measures of cognitive performance administered to two independent cohorts of elderly persons known to be cognitively intact at the time of their initial evaluation. Although the CERAD test battery was not created with specific tests of executive function, our results suggest that the tests we analyzed do contain some information about EF. Our analyses suggested that, for both cohorts, a higher-order latent variable yields a better fit to these data than a first-order model. Both of the multifactor models (with EF as a “causal” higher-order factor, and with EF as a correlated first-order factor) fit the data better than a single-factor model of EF, which did not fit the data in either cohort. These results were observed in two large cohorts of normal elderly who were statistically significantly different in terms of cognitive test scores as well as demographic characteristics. This replication across divergent cohorts, particularly in terms of their respective educational attainments, supports our conclusions that the CERAD battery does contain general information about EF, and that EF can be modeled as a higher-order cognitive faculty. 

Our comparisons of first- and second-order latent variable models suggest that incrementally better fit is obtained with a model hypothesizing EF as a higher-order latent variable, and this was the case whether total score on the MMSE (a general cognitive indicator) was used, whether score on WORLD backwards was separated from score on the other MMSE items, and these were distributed across two factors, or whether we only used the WORLD spelled backwards item on the EF factor. We were unable to statistically compare the models since they are not nested, but the statistical inference is not necessary, since, according to the fit statistic criteria [22], the higher-order model was the best fit to the data in both cohorts, and no matter how the MMSE score was included. 

Importantly, the measurement and structural models supported three latent variables that do not correspond to, for example, three different executive functions (e.g., [8]). The tests that we modeled cannot all be considered to simply represent different executive functions because memory, which is the clear interpretation of the factor with the two memory scores as indicators, is definitely not one of the executive functions [6, 7, 23]. Therefore, our structural model does not represent three different dimensions or components of EF. It is possible that the first-order factor representing “praxis” could be a “lower order” executive function; the amount of variability in “praxis” that is due to the higher-order factor is quite small, in spite of significant pathweights in both of the two cohorts. One critical aspect of this section of the model is that, with the latent variable we interpreted as “executive function” causally influencing the latent variable we interpreted as “praxis”, the latent variable EF is conceptualized as having *indirect *causal influence on the observed “praxis” indicator variables. This feature reinforces the interpretation of the higher-order factor as representing EF, rather than “general cognition”. 

The structural model represents both the higher-order EF factor and the factor we interpreted as “praxis” as causally relevant for the memory factor. Perhaps underlying our results is the fact that in order to perform any task, a variety of functions considered “executive” are needed to a greater or lesser extent [12]. These analyses capitalized on the feature of latent variable modeling that the scores are not expected to represent the underlying latent factor perfectly; our next analytic project is to replicate these models in a cohort with different EF measures and other memory and praxis test scores. Replicating the best-fitting model in two independent cohorts suggests that, with more specific measures of EF in our next study, we should obtain more evidence about whether EF can/should be modeled as a higher-order faculty.

The conceptualization of EF as a higher-order, and/or multidimensional construct is not novel, and yet EF performance is almost universally characterized by the “total score” on one or more tasks specifically designed for either frontal lobe or EF-specific assessment. Our results suggest that, although not specifically designed to assess EF, subtests of the CERAD battery provide theoretically and empirically robust evidence about the nature of EF in elderly adults. They support the conceptualization of EF as multidimensional and hierarchical, with memory and constructional praxis representing the “lower order” dimensions of EF within our models.

It is unclear what the implication is for the clinical day-to-day practice concerning EF evaluation, and although our results were replicated across two independent samples, especially given their baseline cognitive functioning and educational experience differences, it is challenging to conclude that, for example, the tasks we analyzed should be incorporated into EF assessments going forward. The tasks we analyzed, from the CERAD battery, were not specifically designed for the assessment of EF; neuropsychologists are unlikely to adopt CERAD battery tasks for this functionality. However, the results do have implications for the conceptualization of EF in future work, namely, that for research in EF, more complex and multidimensional assessments should be considered. Specifically, the assessment of EF in research settings, and particularly, estimating changes in EF over time, must be conceptualized and considered as more than the simple difference between total scores on EF-specific tasks, over tasks or over time. The definitions of, and tests for, EF vary widely and most authors agree that EF might represent a higher-order cognitive faculty. This work tested the hypothesis explicitly and showed that even with imperfect or incomplete representation of the variety of EF-specific tasks, a latent variable model representing this higher-order function was the best (and a good) fit to the data across independent samples.

In conclusion, the CERAD battery contains some information about executive functioning in elderly persons. We sought statistical evidence for conceptualizing EF as a higher-order function, and this was obtained in independent cohorts. A higher-order structural equation model is a statistical representation that could be a fruitful approach to clarifying the role of EF in other theoretical or experimental settings, or clarifying the assessment of EF in clinical contexts (e.g., [2, 24]). We plan to pursue further evidence of EF as a higher-order faculty and its utility in a clinical context in our future analyses.

## Figures and Tables

**Table 1:** Descriptive statistics of the two cohorts, Mean (SD), or %.

	CERAD (*N* = 460)	EPESE (*N* = 458)
Age*	68.36 (8.0)	79.44 (6.3)
Education*	13.69 (3.2)	8.35 (4.0)
Sex (% female)	65.9%	62.4%,
Race (% white)*	93.0%	40.4%,
Word List Learning (sum of 3 trials)*	20.66 (3.9)	13.59 (4.5) (*N* = 401)
Verbal Fluency*	17.66 (4.9)	12.26 (4.4) (*N* = 389)
Boston Naming*	14.41 (1.2)	11.65 (2.3) (*N* = 389)
Mini-Mental State Exam*	28.75 (1.5)	21.75 (8.2)
MMSE-red*	23.92 (1.3)	21.12 (3.1) (*N* = 373)
WORLD-backwards*	4.86 (0.5)	3.54 (1.6) (*N* = 373)
CP: Circles	1.99 (0.1)	1.96 (0.2) (*N* = 365)
CP: Diamonds*	2.84 (0.4)	2.53 (0.8) (*N* = 365)
CP: Rectangles*	1.99 (0.1)	1.87 (0.5) (*N* = 363)
CP: Cubes*	3.18 (1.2)	1.90 (1.3) (*N* = 359)
Word List Recall*	7.07 (2.0) (*N* = 459)	4.07 (2.2) (*N* = 391)

CP: Constructional Praxis; MMSE-red: MMSE total score without the WORLD backwards item; WORLD backwards: the WORLD backwards item score from the MMSE (the sum of MMSE-red and WORLD backwards gives the total MMSE score).

*Indicates that the difference between these groups is statistically significant (*P* < .001) after Bonferroni correction for multiple (15) comparisons.*N* shows responses less than total sample size.

**Table 2:** Model-data fit two multifactor and one single-factor model of CERAD EF-type tests. Results are shown by study sample and according to whether the total MMSE score, the WORLD backwards item and remaining MMSE total score, or just the WORLD backwards item were included.

Model	Group	Fit Criteria				
		Satorra-Bentler *χ* *2* (df, *P*)	AIC	CFI	SRMR*	RMSEA (90% CI)
Nine scores (total MMSE included on “EF” factor (where >1 factor))						

Higher-order model (one higher-order factor, two first-order factors). Consistent with EF as “higher-order” faculty	CERAD	3.32 (22df, *P* = 1.0)	−40.68	1.0	0.039	0.00 (CI not computed)
	EPESE	8.69 (22df, *P* = .994)	−35.31	1.0	0.040	0.00 (CI not computed)

First-order factors (no higher-order latent factor). Inconsistent with EF as “higher-order” faculty	CERAD	43.27 (24df, *P* = .009)	−4.73	0.958	0.039	0.042 (0.021, 0.062)
	EPESE	32.96 (24, *P* = .10)	−15.04	0.981	0.040	0.033 (0.00, 0.058 )

One-factor model: all test scores reflect a single factor	CERAD	141.91 (27df, *P* < .001)	87.91	0.748	0.078	0.097 (0.081, 0.112)
	EPESE	97.48 (27df, *P* < .001)	43.48	0.848	0.064	0.086 (0.068, 0.105)

Ten scores (MMSE-WORLD on “praxis” factor, WORLD on “EF” factor (where >1 factor))						

Higher-order model (one higher-order factor, two first-order factors). Consistent with EF as “higher-order” faculty	CERAD	18.91 (30df, *P* = .94)	−41.09	1.0	0.052	0.00 (0.00, 0.007)
	EPESE	52.03 (30df, *P* = .008)	−7.97	0.944	0.057	0.048 (0.024, 0.069)

First-order factors (no higher-order latent factor). Inconsistent with EF as “higher-order” faculty	CERAD	65.13 (32df, *P* = .0002)	4.13	0.922	0.052	0.050 (0.033, 0.066)
	EPESE	63.93 (32, *P* < .001)	−0.07	0.918	0.057	0.056 (0.35, 0.075)

One-factor model: all test scores reflect a single factor	CERAD	121.78 (35df, *P* < .001)	51.78	0.814	0.069	0.074 (0.060, 0.088)
	EPESE	90.62 (35df, *P* < .001)	20.62	0.858	0.060	0.070 (0.052, 0.088)

Nine Scores (WORLD on “EF” factor, remainder of MMSE excluded (where >1 factor))						

Higher-order model (one higher-order factor, two first-order factors). Consistent with EF as “higher-order” faculty	CERAD	22.73 (22df, *P* = .42)	−21.27	0.998	0.035	0.009 (0.0, 0.040)
	EPESE	8.69 (22df, *P* = .994)	−24.43	1.0	0.041	0.00 (0.0, 0.040)

First-order factors (no higher-order latent factor). Inconsistent with EF as “higher-order” faculty	CERAD	27.24 (24df, *P* = .294)	−20.765	0.991	0.035	0.017 (0.0, 0.043)
	EPESE	29.60 (24, *P* = .20)	−18.41	0.983	0.041	0.027 (0.00, 0.055)

One-factor model: all test scores reflect a single factor	CERAD	87.86 (27df, *P* < .001)	33.86	0.837	0.070	0.070 (0.054, 0.087)
	EPESE	82.80 (27df, *P* < .001)	28.80	0.832	0.067	0.080 (0.060, 0.099)

*All fit indices have estimation procedures that are robust to distributional and assumptional violations except SRMR. The 90% CI for RMSEA in the higher-order model was not computable for either cohort.

All scores were from the baseline visit. In all models the latent variables derive their scale from standardization of their respective factor variances (set = 1.0).

Fit criteria: Satorra-Bentler *χ* *2*: general robust model fit statistic, with the associated *P*-value for the degrees of freedom shown. Nonsignificant *P*-value suggests “good” fit of model to data. AIC: robust Akaike's Information Criterion; the lower, the better. CFI: Robust Comparative fit index; the closer to 1.0 the better; acceptable models have CFI ≥.95. SRMR: standardized root mean square residuals, the smaller (and <.09) the better. RMSEA: Robust root mean square error of approximation; the closer to zero (and positive) the better; acceptable models have an upper bound on the 90% CI <.06.

**Table 3:** Standardized structural equations (factor loadings only) for observed variables under higher-order EF model (including total MMSE as EF indicator), by study cohort.

Observed variable (indicator)		EPESE cohort path weights*			CERAD cohort path weights	
	EF (2nd order latent variable)	CP or MEM (1st order latent variable)	*R* ^2^, proportion of variance in indicator explained by 1st and 2nd order latent variables	EF (2nd order latent variable)	CP or MEM (1st order latent variable)	*R* ^2^, proportion of variance in indicator explained by 1st and 2nd order latent variables*
Sum of 3 trials (memory)		**.897**	.804		**.845**	.715
Verbal Fluency	**.538**		.289	**.482**		.233
Boston Naming	**.621**		.386	**.481**		.231
MMSE	**.793**		.628	**.724**		.524
CP: circle		**.290**	.084		0.0	0.0
CP: diamond		**.539**	.290		**.493**	.243
CP: rectangle		**.443**	.196		.047	.002
CP: cube		**.572**	.327		**.642**	.412
Delayed Recall		**.728**	.529		**.823**	.677
Factor 2 (CP)	**.808**		.004	**.792**		.280
Factor 3 (MEM)	**.980**	−.279	.911	**1.359**	−.777	.720

*Bentler-Raykov corrected *R* ^2^ coefficients are shown. Bold indicates significant (*P* < .05) pathweight. CP: A factor (latent variable) interpreted as representing constructional praxis. MEM: A factor (latent variable) interpreted as representing memory. EF: A factor (latent variable) interpreted as representing Executive Function.

## Appendix

### Preliminary Exploratory Analyses

There were many different measurement models that could have been selected; so a preliminary exploratory step was performed using TETRAD (v. 4.3.8-6, Spirtes, Scheines, Ramsey & Glymour, 2005; downloaded 20 July 2007 from http://www.phil.cmu.edu/projects/tetrad/. Both data sets, with the three different MMSE configurations, were modeled using the Build Pure Clusters (BPC) and Multiple-Indicators-Model Build (MIMBuild) modules of this program. BPC uses the tetrad difference (determinant of a 2 × 2 submatrix of the overall covariance matrix of the data set; [25]) followed by a partial correlations difference to first obtain evidence of a common cause for the observed variables (tetrad differences = 0) and then to determine that none of the observed variables is that common cause (partial correlations ≠ 0). Together, vanishing tetrad differences plus nonvanishing partial correlations indicate the presence of latent variables that underlie observed variables [26]. Specifically, BPC; finds latent variables that underlie only those observed variables that can be identified as having the single latent variable (representing a “pure” cluster of observed variables) [26]. If BPC finds such pure clusters of observed variables, which form measurement models for their respective latent variables, the TETRAD module MIMBuild will then estimate the relationships between the latent variables identified by BPC, that is, MIMBuild estimates the structural model relating the latents. The structural model will be more, or less, detailed in the sense that the algorithms will simply indicate association (correlation), rather than causality, if insufficient information is present in the data. Thus, the result of the exploratory step with TETRAD analyses provided evidence for the number of latent variables and the observed variables that load solely on each latent variable, plus evidence about the relationships among the latent variables. These functions were run on the scores described in the Materials section, with each of the three different variations on the MMSE scores. These results are not shown, but the majority of the results from this preliminary step supported the measurement models that were fit in the analyses presented in this paper. That is, the exploratory analyses independently reflected the same measurement models underlying the observations from the two cohorts.

## References

[1] Braver TS, West R, Hofer SM, Alwin DF (2008). Working memory, executive control, and aging. *Handbook of Cognitive Aging*.

[2] Luszcz M, Lane AP, Hofer SM, Alwin DF (2008). Executive function in cognitive, neuropsychological, and clinical aging. *Handbook of Cognitive Aging*.

[3] Kemper S, McDowd JM, Hofer SM, Alwin DF (2008). Dimensions of cognitive aging:executive function and verbal fluency. *Handbook of Cognitive Aging*.

[4] Royall DR, Lauterbach EC, Cummings JL (2002). Executive control function: a review of its promise and challenges for clinical research. A report from the committee on research of the American Neuropsychiatric Association. *Journal of Neuropsychiatry and Clinical Neurosciences*.

[5] Alwin DF, Hofer SM, Hofer SM, Alwin DF (2008). Opportunitites and challenges for interdisciplinary research. *Handbook of Cognitive Aging*.

[6] Salthouse TA (2005). Relations between cognitive abilities and measures of executive functioning. *Neuropsychology*.

[7] Rabbitt P, Rabbitt P (1997). Introduction: methodologies and models in the study of executive function. *Methodology of Frontal and Executive Function*.

[8] Miyake A, Friedman NP, Emerson MJ, Witzki AH, Howerter A, Wager TD (2000). The unity and diversity of executive functions and their contributions to complex “frontal lobe” tasks: a latent variable analysis. *Cognitive Psychology*.

[9] Piguet O, Grayson DA, Tate RL (2005). A model of executive functions in very old community dwellers: evidence from the Sydney older persons study. *Cortex*.

[10] Morris JC, Heyman A, Mohs RC (1989). The Consortium to Establish a Registry for Alzheimer’s Disease (CERAD). Part I. Clinical and neuropsychological assessment of Alzheimer’s disease. *Neurology*.

[11] Folstein MF, Folstein SE, McHugh PR (1975). ‘Mini mental state’: a practical method for grading the cognitive state of patients for the clinician. *Journal of Psychiatric Research*.

[12] Thompson JC, Stopford CL, Snowden JS, Neary D (2005). Qualitative neuropsychological performance characteristics in frontotemporal dementia and Alzheimer’s disease. *Journal of Neurology, Neurosurgery and Psychiatry*.

[13] Leonard T, Hsu JSJ (1999). *Bayesian Methods: An Analysis for Statisticians and Interdisciplinary Researchers*.

[14] Cornoni-Huntley J, Blazer DG, Lafferty ME, Everett DF, Brock DB, Farmer ME (1990). *Established Populations for Epidemiologic Studies of the Elderly: Resource Data Book Volume II*.

[15] Fillenbaum GG, Heyman A, Huber MS (1998). The prevalence and 3-year incidence of dementia in older Black and White community residents. *Journal of Clinical Epidemiology*.

[16] Welsh KA, Butters N, Mohs RC (1994). The Consortium to Establish a Registry for Alzheimer's Disease (CERAD). Part V. A normative study of the neuropsychological battery. *Neurology*.

[17] Bennett DA, Schneider JA, Arvanitakis Z (2006). Neuropathology of older persons without cognitive impairment from two community-based studies. *Neurology*.

[18] Kaplan EF, Goodglass H, Weintraub S (1978). *The Boston Naming Test*.

[19] Tombaugh TN, McIntyre NJ (1992). The mini-mental sate examination: a comprehensive review. *Journal of the American Geriatrics Society*.

[20] Rosen WG, Mohs RC, Davis KL (1984). A new rating scale for Alzheimer’s disease. *American Journal of Psychiatry*.

[21] Bentler PM, Wu E (1995). *EQS Structural Equations Program Manual*.

[22] Hu L, Bentler PM (1999). Cutoff criteria for fit indexes in covariance structure analysis. Conventional criteria versus new alternatives. *Structural Equation Modeling*.

[23] Burgess PW, Rabbitt P (1997). Theory and methodology in executive function research. *Methodology of Frontal and Executive Function*.

[24] Ylikoski R, Hänninen T (2003). Assessment of executive function in clinical trials. *International Psychogeriatrics*.

[25] Glymour C, Spirtes P (1988). Latent variables, causal models and overidentifying constraints. *Journal of Econometrics*.

[26] Silva R, Scheines R, Glymour C, Spirtes P (2006). Learning the structure of linear latent variable models. *Journal of Machine Learning Research*.

